# Bilateral lower limb symmetry during sit-to-stand and stand-to-sit tasks in stroke patients with hemiplegia

**DOI:** 10.3389/fneur.2025.1494133

**Published:** 2025-01-29

**Authors:** Meijin Hou, Jian He, Dongwei Liu, Chenyi Guo, Ye Ma, Xiaobo Luo

**Affiliations:** ^1^National Joint Engineering Research Centre of Rehabilitation Medicine Technology, Fujian University of Traditional Chinese Medicine, Fuzhou, China; ^2^Key Laboratory of Orthopaedics and Traumatology of Traditional Chinese Medicine and Rehabilitation (Fujian University of TCM), Ministry of Education, Fuzhou, China; ^3^Research Academy of Grand Health, Faculty of Sports Sciences, Ningbo University, Ningbo, China; ^4^School of Information Technology and Artificial Intelligence, Zhejiang University of Finance and Economics, Hangzhou, China; ^5^Department of Electronic Engineering, Tsinghua University, Beijing, China; ^6^Department of Orthopedics, The 4th Medical Center of the Chinese PLA General Hospital, Beijing, China

**Keywords:** stroke, bilateral, symmetry index, sit-to-stand, stand-to-sit

## Abstract

**Introduction:**

Stroke patients with hemiplegia are at an increased risk of falling during sit-to-stand (Si-St) and stand-to-sit (St-Si) tasks, partly due to impaired bilateral lower limb symmetry. Maintaining symmetrical movement between the limbs in these tasks can help reduce fall incidence.

**Method:**

This study aimed to investigate bilateral lower limb symmetry during Si-St and St-Si tasks in stroke patients with hemiplegia to compare their performance with healthy controls. Thirteen stroke patients and 13 healthy controls participated in the study. Participants were instructed to perform the 30-s chair stand test at their self-selected pace. Kinematic and kinetic parameters were calculated using OpenSim's inverse kinematics and inverse dynamics tools. Bilateral symmetry was quantified using the symmetry index (SI), with an asymmetry threshold set at 10%.

**Results:**

The stroke group exhibited significantly greater lower limb asymmetry in both kinematic and kinetic parameters during Si-St and St-Si tasks compared to the healthy controls, with the kinetic parameters being more pronounced. In the stroke group, notable bilateral asymmetry (SI > 10%) was observed in the ankle joint angle (*P* < 0.05) during both tasks. Furthermore, severe asymmetry (SI > 30%) was identified joint moments across all lower limb joints, vertical ground reaction forces, and medial-lateral center of pressure.

**Discussion:**

These findings highlight the need for targeted rehabilitation programs focusing on improving strength, coordination, and balance. Close monitoring of SI values, particularly for kinetic parameters, is recommended to guide and evaluate the effectiveness of these interventions.

## 1 Introduction

Standing up from a seated position and sitting down from an upright posture, commonly referred to as Sit-to-Stand (Si-St) and Stand-to-Sit (St-Si), are fundamental activities of daily living for humans (1, 2). These movements are frequently performed, with estimates suggesting they occur more than 50 times per day (3, 4). Si-St and St-Si tasks are prerequisite for walking, bed-chair transfers, and maintaining living independence (5, 6). Over recent decades, many studies have examined the biomechanical characteristics of Si-St and St-Si movements in younger and healthy elderly subjects (7–9). These studies have provided normative data on movement patterns and functional performance, which can serve as benchmarks for assessing individuals with movement impairments, such as those following a stroke.

Stroke patients often present various motor deficits, including spasticity, muscle weakness, or hemiplegia (10, 11). These impairments impede stroke patients from performing Si-St and St-Si tasks (12). Instability in the lower limbs, arising from abnormal muscle synergies and weakness, further complicates the execution of these movements (13). To compensate, stroke patients frequently modify their movement strategies (14, 15). These adaptations may include altering chair height, increasing trunk flexion, or changing foot positioning prior to movement (12, 13, 16). Ensuring the safety of stroke patients during Si-St and St-Si tasks is critical, with many relying on their non-paretic leg due to functional limitations on the paretic side (13). However, this compensation can increase the risk of falling (17). Maintaining symmetry between the lower limbs may reduce fall risk during these tasks (18).

While several studies have explored the biomechanical characteristics of Si-St and St-Si tasks in stroke patients using kinematic, kinetic or surface electromyography (EMG) analyses (19–22), relatively few have specifically focused on bilateral lower limb symmetry. (14) identified asymmetry in knee extensor moments during Si-St and St-Si tasks in stroke patients, which was linked to knee extensor weakness and the imbalances in muscle strength on the paretic side (14). Foot position also plays a role in symmetry, with initial positioning affecting vertical ground reaction force (VGRF) symmetry between the limbs (23). For instance, positioning the non-paretic foot behind increases VGRF asymmetry during Si-St or St-Si tasks (12), while positioning the paretic foot behind decrease this asymmetry (24). However, existing research has largely foucued on VGRF, peak joint moments, or symmetry during isolated Si-St task.

Although biomechanical differences in Si-St and St-Si tasks between stroke patients and healthy subjects have identified, few have focused on bilateral lower limb symmetry differences. Understanding these asymmetry is essential for developing effective rehabilitation protocols aim at preventing falls in stroke patients, thereby supporting the recovery of their independence in daily activities. Therefore, this study aims to investigate the bilateral symmetry in both lower limb kinematic and kinetic measures during Si-St and St-Si tasks in stroke patients, with comparison made to healthy controls. We hypothesized that stroke patients with hemiplegia exhibit pronounced bilateral asymmetries in kinematic and kinetic parameters during both Si-St and St-Si tasks, with the asymmetries being more pronounced in the kinetic parameters.

## 2 Methods

### 2.1 Subjects

This study focused on individuals with stroke hemiplegia. Referring to similar research by Frykberg et al. (25), we calculated the required sample size based on a statistical power of 0.8, an effect size (Cohen's d value) of 1.32, an independent sample *t*-test as the statistical method, and a two-sided significant level α = 0.05. Using G*Power software (version 3.1.9.2, Franz Faul, University of Kiel), we determined that a minimum number of 10 participants per group was necessary to achieve adequate statistical power. To account for potential dropouts or missing data, thirteen individuals with stroke hemiplegia (five left paretic and eight right paretic) were recruited as the pathology group. For comparison, thirteen age-matched healthy individuals were also recruited as the control group.

The demographic characteristics of all participants are presented in Table 1. Ethical approval for this study was granted by the Ethics Committee of Shanghai Yangzhi Rehabilitation Hospital (Shanghai Sunshine Rehabilitation Center) under registration number 2020-071. All participants provided written informed consent prior to participation.

**Table 1:** The mean (standard deviation) of the baseline information of all subjects.

	**Stroke group (*N* = 13)**	**Healthy group (*N* = 13)**	***P* valve**
Age (years)	60.46 (5.39)	60.85 (5.93)	0.857
Sex (M:F)	11/2	8/5	0.185
Height (cm)	168.15 (6.88)	165.12 (4.36)	0.057
Weight (kg)	67.08 (10.92)	66.87 (6.31)	0.98
BMI (kg/m2)	23.62 (2.90)	24.51 (1.90)	0.158
Foot length (cm)	23.74 (1.80)	23.81 (1.08)	0.898
Affected side (L/R)	5/8	-	-
MAS (n, 0/1/1^+^/2)	6/4/1/2	-	-
FACS (scores)	4.05 (0.97)	-	-
Brunnstorm (scores)	4.35 (1.06)	-	-

N, number of participants; M, male; F, female; BMI, body mass index; L, left side; R, right side; MAS, Modified Ashworth Scale; FACS, Functional Ambulation Category Scale.

Inclusion criteria for stroke patients were as follows: (1) a confirmed stroke diagnosis via CT or MRI imaging, (2) age over 50 years, (3) a Functional Ambulation Category Scale (FACS) grade between 2 and 5, (4) no history of orthopedic surgery or spasticity treatment within 5 months prior to the experiment, and (5) medical stability, with the ability to complete the required tasks. Healthy subjects were age-matched to the stroke group and had no history of neuromuscular disorders affecting lower limb function. Exclusion criteria for all participants included the presence of other serious illnesses, prior lower limb surgeries, or cognitive impairments.

### 2.2 Experiment protocols

**Figure 1:**
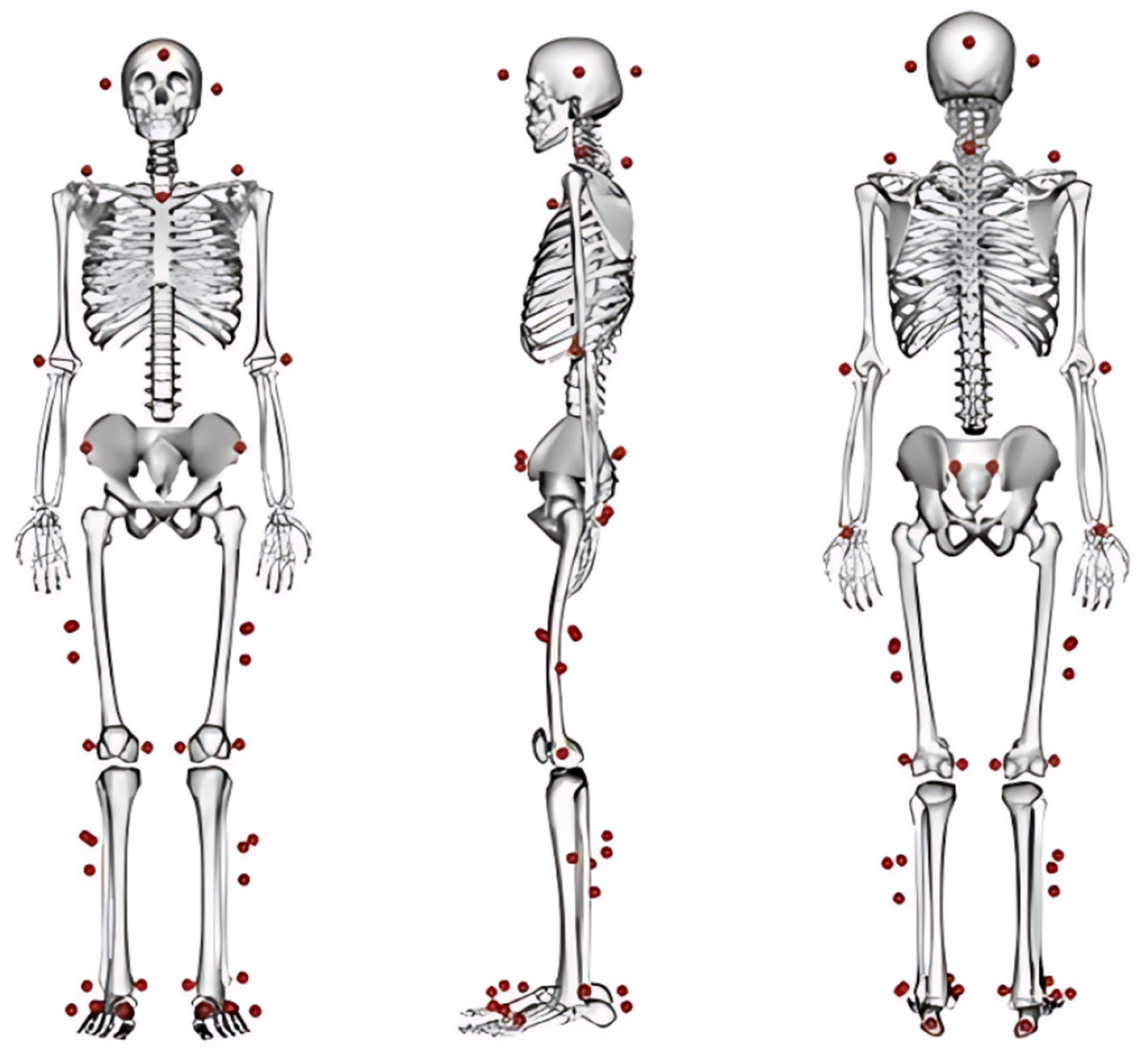
The location of the subject's experimental markers at the time of data collection.

A three-dimensional motion capture system (3DMC) equipped with eight infrared cameras (Vicon Motion Systems Ltd, UK) and two force platforms (AMTI, Watertown, MA, USA) was used to simultaneously record marker trajectories and ground reaction forces. The sampling rates for the marker trajectories and GRF data were 100 Hz and 1,000 Hz, respectively. Reflective markers were placed on key anatomical landmarks, including head, shoulders (bilateral acromion), clavicle, 7th cervical vertebra, hands (lateral epicondyle of humerus, and wrist), pelvis (bilateral anterior and posterior superior iliac spines), thighs (bilateral greater trochanter of the femur, mid-thigh, and medial and lateral epicondyle of femur), shanks (bilateral mid-calf and medial and lateral malleolus), and feet (heel and the first, third and fifth metatarsal bones). A diagram of the marker placement is provided in Figure 1.

Stroke patients were seated barefoot in a chair without a backrest or armrests, positioned with its back edge aligned to the force plates. The chair height was set at 45 cm, corresponding to the typical height for Si-St and St-Si tasks in daily living. Subjects from both group performed two 30-second chair stand tests independently, with a rest period of at least three minutes between trials. Each participant was guided by an experienced physiotherapist to perform the Si-St and St-Si tasks under the following guidelines: (1) Begin in a seated position with an upright posture, feet flat on the force plates, toe pointing forward, and shoulder-width apart. Arms should remain crossed over the chest; (2) Stand fully upright and then sit back down completely; and (3) Perform the movements at their natural speed and as quickly as possible within the 30-second duration. Due to motor dysfunction associated with hemiplegia in some stroke patients, not all individuals could adhere fully to these protocols. In such cases, participants were encouraged to follow the instructions to the best of their ability while prioritizing safety and avoiding undue strain.

Five complete Si-St and St-Si tasks were extracted from the from the recorded 30-second movement cycles for analysis. The start and end points of the Si-St and St-Si tasks were determined by tracking the movement trajectory of reflective markers placed on the bilateral posterior superior iliac spines. The Si-St task was defined as beginning when the reflective marker initiated horizontal forward movement and ending when the marker reached its maximum vertical height, indicating stable standing. The St-Si task began when the marker moved downward from its maximum vertical position and ended when the marker's movement ceased, defined as the point when its velocity consistently remained at zero, indicating stable sitting.

### 2.3 Data processing

The MOtoNMS (Matlab Motion data elaboration Toolbox for NeuroMusculoSkeletal applications) toolbox (26) was used to convert C3D files collected by the 3DMC system into OpenSim-compatible formats. Joint angles and moments for the hip, knee and ankle joints were computed using the inverse kinematics (IK) and inverse dynamics (ID) tools of OpenSim software (Version 3.3, NCSRR, USA). Joint angles were filtered using a zero-lag fourth-order low-pass Butterworth filter with a cutoff frequency of 6 Hz (27). Ground reaction force (GRF) and center of pressure (COP) data were also filtered using a a zero-lag fourth-order Butterworth low-pass filter with a cut-off frequency of 20 Hz (27). All data were then time-normalized to 100% of the motion cycle using a custom Matlab script. GRF and joint moments were normalized to body weight, while COP data were normalized to “foot length,” defined as the distance from the heel to the first metatarsal.

Symmetry measurements were quantified by comparing the left and right limb characteristics (28). For each subject, bilateral variables of interest were categorized as either “greater” or “lesser” (29) (see Equation 1).


(1) $$\begin{array}{l} Symmetry\;index\left(\%\right)=100\times \left(1-\frac{lesser\;values}{greater\;values}\right) \end{array}$$

Equation 1 is derived from a previously established limb symmetry index (30). According to this formula, a 50% asymmetry between limbs is presented if the values of the larger variables is double that of the smaller one, while perfect symmetry is indicated when the values are identical (29). In this study, an SI of 10% was used to distinguish between symmetry and asymmetry (29, 31, 32).

For the temporal parameters, we calculated the time to reach the maximum joint moment, joint angle, vertical ground reaction force (VGRF), and the total durations of Si-St and St-Si movements. For the bilateral SI values of kinematic and kinetic parameters in the stroke and control groups, we extracted the mean, initial, peak, and final phases SI of the hip, knee, and ankle joint angles and moments during Si-St and St-Si movements. Additionally, the bilateral SI of joint range of motion (ROM), defined as the maximum joint angle minus minimum joint angle, was also calculated. We also extracted the mean, initial, peak, and final phases of bilateral SI in VGRF and COP in the anterior-posterior (AP) and medial-lateral (ML) directions.

### 2.4 Statistical analysis

The Shapiro-Wilk test was conducted to assess the normality of the data distribution. For variables that followed a normal distribution, a paired-sample *t*-test was applied to compare spatial-temporal differences between limbs, while an independent sample *t*-test was used to evaluate the differences in kinematic and kinetic SI values between the stroke and control groups. For parameters that did not meet the normality assumption, the Wilcoxon Signed Rank Test was employed to compare bilateral spatial-temporal differences, and the Mann-Whitney U test was used for between-group comparisons. A significance level of 0.05 was set for all statistical tests. Data are presented as mean ± standard deviation (SD) in both the text and tables. Statistical analyses were conducted using SPSS software version 26 (IBM/SPSS Inc., Armonk, NY, USA).

## 3 Results

### 3.1 Bilateral lower limbs symmetry in joint angles

**Figure 2:**
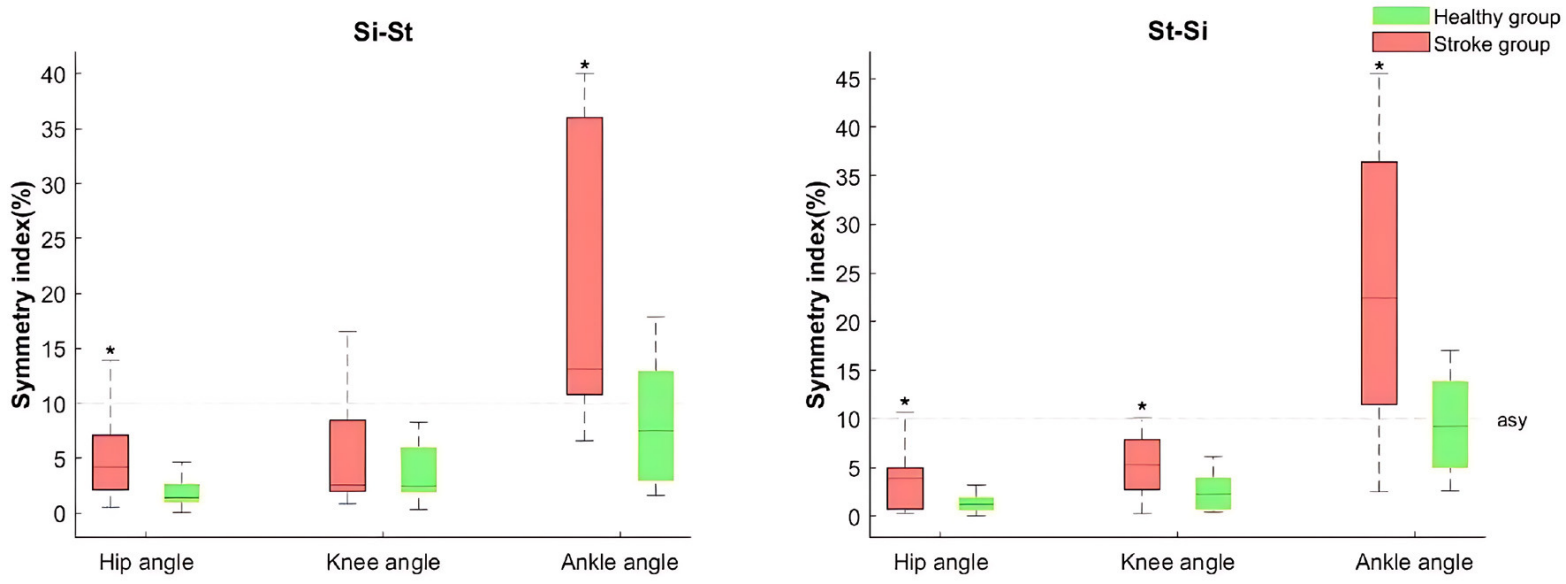
Boxplots of the joint angle symmetry index (SI) of the lower limb for both stroke and healthy groups during sit-to-stand (Si-St) and stand-to-sit (St-Si) tasks. The boxes represent the interquartile range, with medians indicated by horizontal line, and whiskers denoting the upper and lower 25% of the distribution. The transverse dotted lines indicate the asymmetric baseline. An asterisk (*) symbol denotes a significant difference compared to healthy group (*P* <0.05).

Figure 2 illustrates the mean SI values for joint angles in stroke and healthy subjects during Si-St and St-Si tasks. Table 2 provides a detailed breakdown of the mean values, isolated movement phases (initial, peak, and final), and the range of motion (ROM) during the Si-St and St-Si tasks.

**Table 2:** The SI (%) of joint angle between stroke and healthy group during Si-St and St-Si tasks.

**Tasks**	**Phases**	**Stroke group**			**Healthy group**		
		**Hip**	**Knee**	**Ankle**	**Hip**	**Knee**	**Ankle**
Si-St	Mean	5.35 (4.21)^*^	6.07 (6.02)	20.06 (12.72)^*^	1.91 (1.46)	3.53 (2.39)	8.70 (5.81)
	Initial	3.48 (2.56)^*^	3.29 (3.07)	17.29 (14.16)	1.49 (1.19)	1.83 (2.06)	25.65 (27.27)
	Peak	3.22 (2.57)^*^	3.29 (3.06)	12.66 (8.13)	1.44 (0.94)	1.84 (2.06)	21.97 (24.10)
	Final	23.24 (17.94)	24.96 (18.19)	32.03 (27.26)	25.77 (24.96)	26.81 (30.15)	27.06 (18.88)
	ROM	4.97 (4.94)	5.37 (6.41)	17.59 (13.08)^*^	2.20 (1.63)	3.34 (2.52)	12.13 (7.37)
St-Si	Mean	4.36 (4.43)^*^	6.87 (5.77)^*^	22.67 (14.20)^*^	1.50 (1.42)	2.42 (1.81)	10.44 (6.92)
	Initial	22.69 (17.56)	26.36 (17.35)	35.45 (24.63)	26.82 (28.82)	21.61 (23.88)	25.27 (18.25)
	Peak	3.02 (2.81)	3.61 (4.02)	15.76 (9.40)	1.46 (1.00)	1.64 (1.53)	21.55 (20.98)
	Final	3.52 (3.11)	3.62 (4.02)	20.49 (14.55)	1.79 (1.29)	1.65 (1.53)	26.35 (25.36)
	ROM	4.36 (4.43)	6.87 (5.77)*	22.67 (14.20)^*^	2.62 (1.95)	3.36 (3.20)	11.53 (6.68)

Values are expressed as mean (SD).

Si-St, Sit-to-stand; St-Si, stand-to-sit; ROM, range of motion.

^*^ symbol indicates significant difference with the healthy group (*P* < 0.05).

In the stroke group, the SI values (including the median, mean, initial, peak, final values and ROM) in ankle angles during both tasks exceeded 10% with the mean SI for the ankle angles being significantly (*P* < 0.05) higher compared to the healthy group, indicating pronounced asymmetry (see Figure 2 and Table 2). Notably, healthy individuals also demonstrated asymmetry in ankle joint angles during both tasks, although no significant differences were found between limbs (*P* >0.05).

For hip and ankle angles, significant differences (*P* < 0.05) were observed in the mean SI values for hip angle during the Si-St task, and for hip and knee angles during the St-Si task when comparing the stroke group to the healthy group. Furthermore, the stroke group exhibited SI values for hip and knee angles exceeding 20% in the final phase of the Si-St task and the initial phase of the St-Si task, though these differences were not statistically significant (*P* >0.05) compared to the healthy group.

### 3.2 Bilateral lower limbs symmetry in joint moments

**Figure 3:**
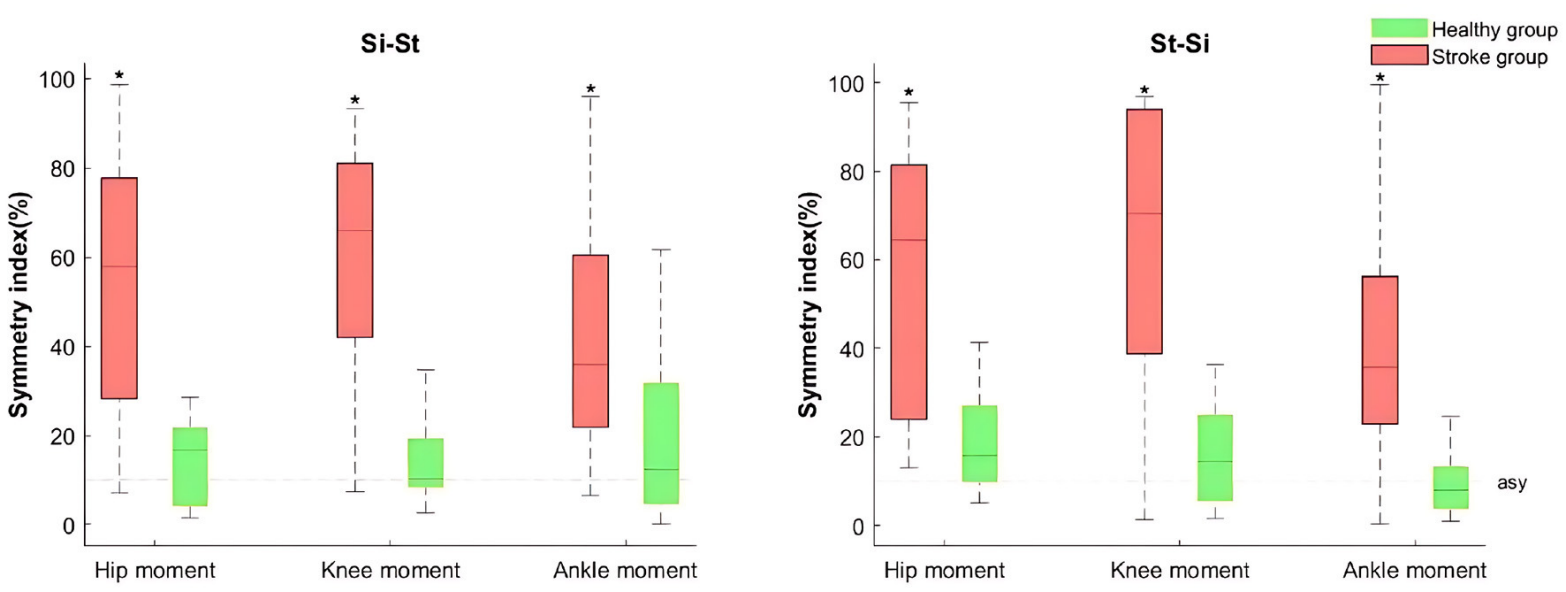
Boxplots of lower limb joint moment SI for the stroke and healthy groups during Si-St and St-Si tasks. The Boxes represent the interquartile range, with medians indicated by the horizontal lines, and whiskers representing the upper and lower 25% of the distribution. The dashed lines indicate the asymmetry baseline. Asterisks (*) denote significant differences compared to the healthy group (*P* <0.05).

The Median and mean SI values of hip, knee and ankle joint moments in the stroke group during both Si-St and St-Si tasks exceeded 10% and 50%, receptively (see Figure 3). Notably, the SI values for all lower limb moments exceeded 20% across all phases of the tasks, indicating substantial bilateral asymmetry in joint moments. Both the mean and peak SI values for hip, knee, and ankle joint moments were significantly different (*P* < 0.05) between the stroke and healthy groups during Si-St and St-Si tasks. The SI for ankle moments across all phases (initial, peak, and final) showed significant differences (*P* < 0.05) between the two groups during the St-Si task. Furthermore, the initial SI of hip and ankle joint moments during the St-Si task were significantly higher (*P* < 0.05) in the stroke group compared to the healthy group.

### 3.3 Bilateral symmetry in GRF and COP

**Figure 4:**
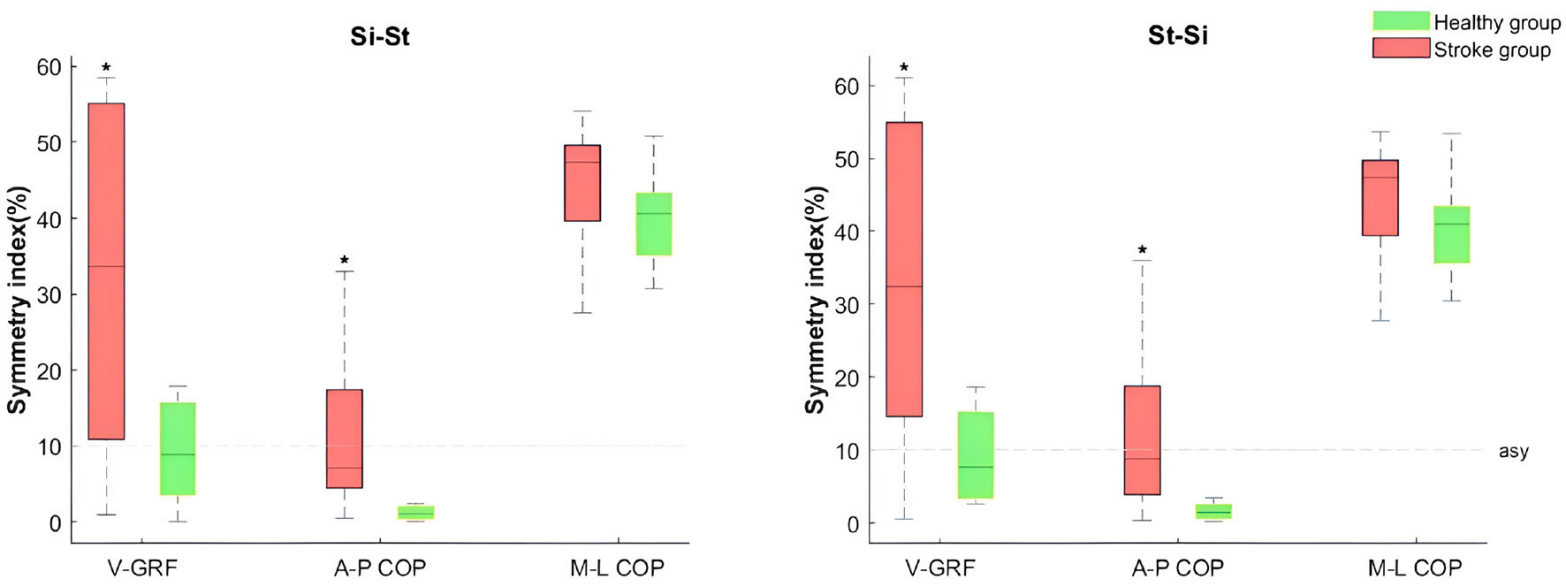
Boxplots of lower limb mean vertical ground reaction force (VGRF), anterior-posterior center of pressure (A-P COP), and medial-lateral center of pressure (M-L COP) SI between the stroke and healthy groups during Si-St and St-Si tasks. The boxes represent the interquartile range, with medians indicated by a horizontal line, and whiskers representing the upper and lower 25% of the distribution. The dashed lines indicate symmetry baselines. The ^*^ symbol denotes significant differences compared to the healthy group (*P* <0.05).

The stroke group exhibited asymmetry in VGRF and ML-COP between both sides, with SI values exceeding 20% during the Si-St and St-Si tasks (Table 4). The median SIs for VGRF and ML-COP in the stroke group were greater than 10% (Figure 4). Additionally, the SI for VGRF and ML-COP during Si-St and St-Si tasks showed significant difference (*P* < 0.05) between the two groups. For COP, the SI of the average and initial phase AP-COP exceeded 10% in the stroke group during both tasks. The SI of the AP-COP in the stroke group was significantly different (*P* < 0.05) compared with the healthy group for most phases (except the peak phase during Si-St task) during Si-St and St-Si tasks.

## 4 Discussion

This study investigated the temporal characteristics, and bilateral symmetry characteristics of kinematic and kinetic parameters during sit-to-stand (Si-St) and stand-to-sit (St-Si) tasks in stroke patients, and compared these measures with healthy controls.

Si-St and St-Si movements are bilateral tasks that require coordinated manipulation of both the right and left limbs to successfully complete the actions (14). Achieving motor symmetry between the bilateral lower limbs is crucial for efficient task performance. Symmetry is often defined as the perfect agreement of the external kinetics and kinematics of the left and right legs (33).

Simply distinguishing between symmetry and asymmetry based on statistical differences in movement variables may be overly simplistic. There remains no universally accepted standard for determining when symmetry in Si-St and St-Si tasks has improved or returned to the levels observed in healthy individuals, as noted in the literature on asymmetry (12, 15, 34). Discrepancies in asymmetry research often arise due to varying definitions of asymmetry, differing methodologies, and the use of different variables and formulas for calculating asymmetry. (29) proposed that the Symmetry Index (SI) formula is an appropriate method for assessing bilateral symmetry and asymmetry during Si-St and St-Si tasks. Additionally, the initial and final phases of both the Si-St and St-Si movements have been shown to be particularly sensitive in distinguishing the motor functions of individuals with varying abilities (35), The SI characteristics during these phases have been further investigated to provide insights into functional impairments.

The results of our study support our hypothesis, revealing significantly larger SI values in both kinematic and kinetic parameters in comparison with healthy controls (*P* < 0.05). Pronounced bilateral asymmetry (SI >10%) was observed in the mean and ROM of ankle angles, joint moments at the hip, knee and ankle joints, vertical GRF, anterior-posterior COP and initial medial-lateral COP during both tasks. Notably, the asymmetries in kinetic parameters were more substantial and significant compared to those in kinematic parameters.

Stroke patients exhibited symmetrical movements (SI < 10%) in most lower limb joint angles, except during the initial and final phases, where a few exceptions occurred. Ankle asymmetry suggests inconsistent ankle mechanics during Si-St and St-Si tasks, potentially caused by restricted mobility or excessive dorsiflexion on the paretic side. In contrast, SI values exceeding 30% were identified in kinetic parameters across both tasks. This finding aligns with Lomaglio and Eng (15), who reported ankle moments as the most asymmetric parameter during Si-St movements in stroke patients. Our results further revealed more severe joint moment asymmetry (SI between 30% to 65%) in stroke patients compared to healthy individuals.

This asymmetry likely stems from significant weakness in hip, knee, and ankle flexors and extensors on the affected side, particularly after the Si-St off-seat phase (reaching peak moment) and before the St-Si contact-seat phase (approaching peak moment). To ensure smooth execution of the task, the healthy limb compensated for the affected side, bearing increased joint loads. Additionally, foot placement strategies influence joint moment asymmetry in stroke patients. Asymmetry in knee moments decreases when the affected foot is positioned behind and increases when the healthy foot is placed behind (14, 15).

Some degree of asymmetry (SI >10%) was also detected in ankle joint angles, moments, vertical GRF, and medial-lateral COP in specific movement phases among healthy controls (see Tables 3, 4). This highlights that asymmetry, while more pronounced in stroke patients, can also occur in healthy individuals under certain conditions (36).

**Table 3:** The SI (%) of joint moment between the stroke and healthy group during Si-St and St-Si tasks.

**Tasks**	**Phases**	**Stroke group**			**Healthy group**		
		**Hip**	**Knee**	**Ankle**	**Hip**	**Knee**	**Ankle**
Si-St	Mean	53.63 (31.85)^*^	59.16 (28.74)^*^	40.61 (25.87)^*^	14.03 (9.24)	14.39 (9.82)	19.95 (18.96)
	Initial	45.64 (26.27)	64.34 (26.74)^*^	47.51 (29.50)	56.58 (30.19)	34.78 (25.05)	39.15 (28.03)
	Peak	43.50 (22.00)^*^	44.96 (25.59)^*^	39.29 (29.66)*	13.11 (7.54)	10.18 (8.29)	19.75 (15.61)
	Final	72.28 (22.40)^*^	63.84 (30.73)	39.19 (31.00)	31.91 (18.83)	40.46 (34.06)	20.10 (15.39)
St-Si	Mean	57.37 (29.15)^*^	65.11 (32.10)^*^	40.28 (26.19)^*^	18.32 (11.02)	15.03 (11.50)	9.26 (7.00)
	Initial	54.33 (29.18)^*^	53.24 (28.73)	40.35 (30.45)^*^	25.19 (21.97)	33.57 (20.91)	12.56 (14.07)
	Peak	48.00 (25.84)^*^	49.18 (26.60)^*^	38.43 (30.20)^*^	20.00 (13.20)	14.41 (8.51)	9.45 (9.41)
	Final	34.60 (30.72)	50.08 (32.79)	49.49 (31.56)^*^	34.68 (27.13)	29.26 (17.55)	26.29 (22.57)

Values are expressed as mean (SD).

Si-St, Sit-to-stand; St-Si, stand-to-sit.

^*^ symbol indicates significant difference with the healthy group (*P* < 0.05).

**Table 4:** The SI (%) of VGRF, AP-COP and ML-COP between the stroke and healthy group during Si-St and St-Si tasks.

**Tasks**	**Phases**	**Stroke group**			**Healthy group**		
		**VGRF**	**AP-COP**	**ML-COP**	**VGRF**	**AP-COP**	**ML-COP**
Si-St	Mean	31.87 (22.42)*	12.30 (11.35)^*^	44.26 (8.24)	9.22 (6.44)	1.78 (2.19)	39.99 (6.23)
	Initial	23.97 (13.50)	12.00 (10.07)^*^	45.27 (7.70)^*^	14.95 (10.94)	1.69 (1.45)	38.71(5.56)
	Peak	28.70 (23.27)^*^	8.87 (12.01)	43.71 (7.84)	8.04 (4.53)	5.70 (8.68)	42.93(8.57)
	Final	29.62 (21.46)^*^	9.92 (8.72)^*^	45.22 (7.42)	10.53 (7.55)	2.35 (1.95)	42.60 (6.42)
St-Si	Mean	32.51 (21.60)^*^	12.28 (11.56)^*^	44.07 (8.14)	9.06 (6.19)	1.52 (1.08)	40.37 (6.58)
	Initial	33.07 (18.24)^*^	10.48 (9.86)^*^	45.0 (7.69)	11.22 (8.60)	1.75 (1.52)	42.32 (6.66)
	Peak	30.80 (21.29)^*^	8.25 (9.71)^*^	43.62 (7.86)	10.60 (6.19)	1.53 (1.28)	41.04 (6.99)
	Final	24.87 (13.05)	9.22 (9.54)^*^	45.17 (8.02)	16.87 (13.46)	2.42 (1.46)	40.85 (6.40)

Values are expressed as mean (SD).

Si-St, Sit-to-stand; St-Si, stand-to-sit; VGRF, vertical ground reaction force; AP-COP, anterior-posterior center of pressure; ML-COP, medial-lateral center of pressure.

^*^ symbol indicates significant difference with the healthy group (*P* < 0.05).

Stroke patients with hemiplegia often experience postural control deficits due to hemiplegic weakness, leading to an asymmetrical pattern when standing up from a chair (5). Although adjustments to foot placement and chair height can help reduce this asymmetry (12, 13, 24), stroke patients often struggle to maintain these adjusted postures in daily activities. Previous studies have shown that the paretic leg supports only 37.9% of the body weight, compared to 50.5% and 49.5% distributed equally across both legs in healthy controls (37). Our findings support this, as bilateral vertical GRF revealed asymmetry (SI >20%) in the stroke group during both the Si-St and St-Si tasks, while the healthy group displayed symmetry (SI < 10%) in weight distribution between both legs.

Regarding balance and postural control, the stroke group displayed slight bilateral asymmetry in AP-COP (SI between 8% to 13%) during both tasks. Both the stroke and healthy groups showed significant bilateral asymmetry in ML-COP (SI >30%), indicating compromised postural control and stability in the ML direction. The asymmetry in GRF and ML-COP in the stroke group suggests that they are using a compensatory movement strategy, where the non-paretic leg compensates for the weaker paretic side. Additionally, muscle weakness and impaired motor control on the paretic side likely force the non-paretic leg to take on more of the load during the Si-St and St-Si tasks (38, 39).

Increased SI values indicate an imbalance in force production between the paretic and non-paretic limbs, along with impaired muscle control, balance, and movement coordination. Analyzing SI values helps guide targeted rehabilitation strategies for individuals with stroke hemiplegia. These strategies may include resistance training to improve paretic limb strength, neuromuscular re-education to enhance motor control, and balance training to address postural stability. Additionally, bilateral coordination exercises can promote symmetry and improve functional performance in tasks such as sit-to-stand and stand-to-sit movements. Monitoring changes in SI values over the course of rehabilitation provides valuable feedback on intervention efficacy and recovery progress.

The limitations of our study should be acknowledged. First, the effect of varying levels of disability on bilateral lower limb symmetry was not considered. Future research should involve a larger sample size and examine the influence of different levels of disability and spasticity on symmetry during Si-St and St-Si movements in stroke patients. Second, the impact of trunk kinematics and postural control strategies on lower limb symmetry needs further investigation.

## 5 Conclusion

This study is one of the few to specifically quantify the bilateral symmetry of kinematic and kinetic parameters during sit-to stand (Si-St) and stand-to-sit (St-Si) tasks in stroke patients with hemiplegia, and to compare these measures with healthy individuals. Bilateral asymmetry was particularly pronounced in stroke patients, especially regarding ankle dorsiflexion angle, hip, knee, and ankle joint moments, as well as vertical GRF and COP in the medial-lateral direction. The SI values for these parameters in stroke patients were consistently higher than in the healthy group. These findings suggest that targeted resistance, balance and coordination training, targeted to improve the bilateral symmetry in both kinematic and kinetic parameters, may enhance the functions of Si-St and St-Si transitions and reduce fall risks. Monitoring changes in SI values over the course of rehabilitation provides valuable feedback on intervention efficacy and recovery progress.

## Data Availability

The datasets presented in this study can be found in online repositories. The names of the repository/repositories and accession number(s) can be found below: https://www.scidb.cn/s/bEvIZv.
